# Long non‐coding RNA F11‐AS1 inhibits HBV‐related hepatocellular carcinoma progression by regulating NR1I3 *via* binding to microRNA‐211‐5p

**DOI:** 10.1111/jcmm.14881

**Published:** 2019-12-27

**Authors:** Yibin Deng, Zhongheng Wei, Meijin Huang, Guidan Xu, Wujun Wei, Bin Peng, Shunqiang Nong, Houji Qin

**Affiliations:** ^1^ Clinic Medicine Research Center of Hepatobiliary Diseases The Affiliated Hospital of Youjiang Medical College for Nationalities Baise China; ^2^ Department of Infectious Diseases The Affiliated Hospital of Youjiang Medical College for Nationalities Baise China; ^3^ Centre for Medical Laboratory Science The Affiliated Hospital of Youjiang Medical College for Nationalities Baise China; ^4^ Department of Oncology The Affiliated Hospital of Youjiang Medical College for Nationalities Baise China

**Keywords:** hepatitis B virus, hepatocellular carcinoma, long non‐coding RNA F11‐antisense 1, microRNA‐211‐5p, nuclear receptor constitutive androstane receptor

## Abstract

Long non‐coding RNAs (lncRNAs) could regulate growth and metastasis of hepatocellular carcinoma (HCC). In this study, we aimed to investigate the mechanism of lncRNA F11‐AS1 in hepatitis B virus (HBV)–related HCC. The relation of lncRNA F11‐AS1 expression in HBV‐related HCC tissues to prognosis was analysed in silico. Stably HBV‐expressing HepG2.2.15 cells were established to explore the regulation of lncRNA F11‐AS1 by HBx protein, as well as to study the effects of overexpressed lncRNA F11‐AS1 on proliferation, migration, invasion and apoptosis in vitro. Subsequently, the underlying interactions and roles of lncRNA F11‐AS1/miR‐211‐5p/NR1I3 axis in HBV‐related HCC were investigated. Additionally, the influence of lncRNA F11‐AS1 and miR‐211‐5p on tumour growth and metastasis capacity of HepG2.2.15 cells were studied on tumour‐bearing nude mice. Poor expression of lncRNA F11‐AS1 was correlated with poor prognosis in patients with HBV‐related HCC, and its down‐regulation was caused by the HBx protein. lncRNA F11‐AS1 was proved to up‐regulate the NR1I3 expression by binding to miR‐211‐5p. Overexpression of lncRNA F11‐AS1 reduced the proliferation, migration and invasion, yet induced apoptosis of HepG2.2.15 cells in vitro, which could be abolished by overexpression of miR‐211‐5p. Additionally, either lncRNA F11‐AS1 overexpression or miR‐211‐5p inhibition attenuated the tumour growth and metastasis capacity of HepG2.2.15 cells in vivo. Collectively, lncRNA F11‐AS1 acted as a modulator of miR‐211‐5p to positively regulate the expression of NR1I3, and the lncRNA F11‐AS1/miR‐211‐5p/NR1I3 axis participated in HBV‐related HCC progression *via* interference with the cellular physiology of HCC.

## 1 INTRODUCTION

The leading cause of hepatocellular carcinoma (HCC) is infection caused by hepatotropic viruses such as hepatitis B virus (HBV), HCV and HDV, among which HBV infection serves as the main trigger accounting for approximately 54% of all liver cancer cases.^1^ The mechanisms that contribute to HBV‐induced HCC include deregulated control of cellular proliferation and apoptosis, which may be partially attributed to abnormal alterations in certain gene expression such as hepatitis B virus X (HBx) protein.^2^ Currently, various therapeutic options are applied for HCC, such as chemotherapeutics and miRNA‐based therapies combining surgery.^3^ Additionally, liver transplantation and resection are considered as the best approaches to treat HCC and to deliver long‐term survival for the early‐diagnosed HCC patients. However, these methods are not suitable for advanced HCC patients, owing to disappointing 5‐year survival rates.^4^ Hence, it is trivial to attain a better and deeper understanding of the pathophysiology of HCC to effectively treat this liver cancer.

Recently, dysregulation of long non‐coding RNAs (lncRNAs) has been highlighted to share association with tumorigenesis and metastasis of HCC.^5^ Particularly, lncRNA F11‐antisense 1 (lncRNA F11‐AS1) has been uncovered to be implicated in the progression of some carcinomas. For instance, down‐regulated levels of lncRNA F11‐AS1 have been documented in pancreatic ductal adenocarcinoma (PDAC) cells, suggesting that its misregulation might exert critical roles in PDAC.^6^ In addition, lncRNA F11‐AS1 is also poorly expressed in ovarian cancer; moreover, differential expression of lncRNA F11‐AS1 in cell lines that exhibited platinum‐based drug resistance or non‐resistance implied that lncRNA F11‐AS1 could be regarded as a prognostic marker to predict the incidence of platinum‐based chemoresistance.^7^ All these previous findings and results indicate the prognostic and therapeutic values of lncRNA F11‐AS1 in multiple cancers; however, the exact role of lncRNA F11‐AS1 in HCC has not been investigated yet.

More recently, emerging evidence has demonstrated that lncRNAs can function as a competing endogenous RNA (ceRNA) for specific miRNAs and regulate their function and downstream targets of miRNA to influence the development of HCC.^8^, ^9^ For example, lncRNA F11‐AS1 is reported to bind to miR‐3146 to affect the HCC progression.^10^ microRNA‐211 (miR‐211) is closely related to the progression and development of human cancers, and lncRNA SNHG15 promotes the occurrence and progression of lung cancer by enhancing the proliferative and migration capacity of lung cancer cells through binding to miR‐211‐3p.^11^ In oral squamous cell carcinoma, miR‐211‐3p directly suppresses the transcription factor 12, which regulates the activities of cancer‐related suppressors in tumour cells.^12^ In silico analysis and luciferase experiment in the current study confirmed that lncRNA F11‐AS1 could potentially bind to miR‐211‐5p and nuclear receptor subfamily 1, group I, member 3 (NR1I3), also known as CAR, was a target gene of miR‐211‐5p. The functionality of NR1I3, an important regulator of drug metabolism and cancer development, is mediated *via* modulation of transcription of target genes.^13^ Therefore, we proposed a hypothesis that the interactions among lncRNA F11‐AS1/miR‐211‐5p/NR1I3 axis might be involved in tumorigenesis of HBV‐related HCC, and the subsequent experiments in the current study were performed to study the effects of this axis on the physiological functions of stably HBV‐expressing HepG2.2.15 cells.

## 2 MATERIALS AND METHODS

### 2.1 Ethics statement

The current study was approved by the Ethics Committee of the Affiliated Hospital of Youjiang Medical College for Nationalities. Signed informed consent was obtained from all participating patients prior to the study. All animal procedures were conducted in accordance with the National Institutes of Health Guide for the Care and Use of Laboratory Animals, and utmost humane care was exercised while dealing with the animals based on the guidelines approved by the Animal Ethics Committee of the Affiliated Hospital of Youjiang Medical College for Nationalities.

### 2.2 Sample collection

HCC tissues and normal adjacent tissues (at least 5 cm from the edge of the tumour) were collected from patients who were diagnosed with HCC between January 2010 and January 2013 at the Affiliated Hospital of Youjiang Medical College for Nationalities. None of the included patients had undergone any radiotherapy and chemotherapy prior to the operation. Patients were followed up from January 2015 to January 2018. The clinical data of 73 HCC patients including 45 patients with HBV positive (+) and 28 patients with HBV negative (−) are shown in Table 1. The tissue samples were frozen in liquid nitrogen and stored in a −80°C refrigerator for subsequent use.

**Table 1:** The correlations of lncRNA F11‐AS1 expression and clinicopathological features of HCC patients

Variables	n	lncRNA F11‐AS1		*P*‐value
		High expression (n = 25)	Low expression (n = 48)	
Age (years)				
<55	44	16	28	0.828
≥55	29	9	20	
Gender				
Male	51	17	34	0.985
Female	22	8	14	
HBsAg				
Positive (+)	45	10	35	0.013
Negative (−)	28	15	13	
AFP (ng/mL)				
>200	45	18	27	0.289
≤200	28	7	21	
Tumour size (cm)				
>5	35	6	29	0.007
≤5	38	19	19	
Tumour number				
Solitary	35	17	18	0.026
Multiple	38	8	30	
TNM stage				
I‐II	38	18	20	0.027
III‐IV	35	7	28	

Note

Data were analysed by chi‐square test.

Abbreviations: AFP, alpha‐fetoprotein; TNM, tumour node metastasis; lncRNA F11‐AS1, long non‐coding RNA F11‐antisense 1.

### 2.3 Cell culture

The human normal liver HL‐7702 cell line, HCC cell lines (Huh7, HepG2, HepG2.2.15 and SMMC‐7721) and HEK‐293T cell line were purchased from the American Type Culture Collection (ATCC; https://www.atcc.org/). All the above‐mentioned cell lines were separately cultured in Dulbecco's modified Eagle's medium (DMEM, D0819) supplemented with 10% foetal bovine serum (FBS, 10100147). Next, 100 U/mL penicillin/streptomycin (15 140 122) was added to the medium, and the cells were cultured with 5% CO_2_ in air at 37°C. Stably HBV‐expressing HepG2.2.15 cells were incubated in minimum essential medium (MEM)‐α medium (M8042) containing 10% FBS, and additionally supplemented with 100 U/mL penicillin/streptomycin, 200 μg/mL G418 (1013102700) and 2 mmo/L l‐glutamine. Then, the cells were maintained under a condition of 5% CO_2_ at 37°C.^14^ The cell culture medium and reagents used in this part were all purchased from Gibco BRL/Invitrogen. Expression of lncRNA F11‐AS1 in each cell line was determined by reverse transcription‐quantitative polymerase chain reaction (RT‐qPCR), and the cell line exhibiting the lowest lncRNA F11‐AS1 expression was selected for subsequent experimentation. The experiments were repeated 3 times to obtain the mean value.

### 2.4 Cell transfection

Cell transfection was conducted according to the instructions of Lipofectamine 2000 transfection kit (11668019; Invitrogen). A total of 1 × 10^6^ cells were treated with 50 μg plasmids or lncRNA F11‐AS1 overexpression plasmid (oe‐lncF11‐AS1), miR‐211‐5p mimic, miR‐211‐5p inhibitor, oe‐NR1I3 and their corresponding negative controls (oe‐NC and miR‐NC), mixed well and later incubated for 6 h at 37°C. Afterwards, the culture medium was renewed with complete medium, following incubation for another 24‐48 hours. RNA content extraction from cells was performed in order to assess the transfection efficiency. All the plasmids used in this study were constructed by Shanghai Sangon Biotechnology Co., Ltd.

### 2.5 Flow cytometry

After transfection for 48 hours, the cells were treated with ethylenediaminetetraacetic acid (EDTA)‐free 0.25% trypsin (YB15050057; Shanghai Yubo Biotechnology Co., Ltd.) and collected in a flow tube. The cells were then centrifuged with the supernatant discarded. Subsequently, cell apoptosis assay was performed with Annexin V‐fluorescein isothiocyanate (FITC)/propidium iodide (PI) apoptosis detection kits (K201‐100; BioVision) according to the manufacturer's instructions. The cells were excited at 488 nm, and FITC fluorescence was detected using a 525‐nm band‐pass filter, while PI fluorescence was detected using a 620‐nm band‐pass filter. The apoptotic cells were calculated by the number of positive FITC cells minus positive PI cells. The experiments were repeated 3 times to obtain the mean value.

### 2.6 RNA isolation and quantitation

Total RNA content extraction from tissues or cells after 36‐hours transfection was performed according to the manufacturer's protocols provided by the TRIzol reagent (10296010; Invitrogen). All the primers (Table 2) were synthesized by BGI Biotech Co., Ltd. (Beijing, China). For RT‐qPCR of miRNAs, 100 ng of total RNA was reverse‐transcribed and subjected to TaqMan^TM^ MicroRNA assay (4366596; Applied Biosystems, Inc). Subsequently, the expression of miR‐211‐5p was determined by qPCR with a TaqMan^TM^ MicroRNA assay kit (4427975; Applied Biosystems, Inc) and standardized using U6. For RT‐qPCR of mRNAs, cDNA synthesis was performed with the total RNA using EasyScript First‐Strand cDNA Synthesis SuperMix (AE301‐02; Beijing TransGen Biotech Co., Ltd.) and standardized using housekeeping coding gene glyceraldehyde 3‐phosphate dehydrogenase (GAPDH). Subsequently, the quantitative analysis of mRNA expression was conducted according to the instructions of SYBR^®^ Premix Ex TaqTM II kit (RR820A; TaKaRa) on an ABI7500 real‐time PCR instrument (Applied Biosystems). Finally, the fold changes were calculated by means of relative quantification (2^−∆∆Ct^). The experiments were repeated 3 times to obtain the mean value.

**Table 2:** Primer sequences for RT‐qPCR

Gene	Sequence (5'–3')
miR‐211‐5p	F: CTCGAGTAACCGTATTGTTCGCGTCATGCCAGCA
	R: GCGGCCGCCAGACCATGTGTCCCATTTG
U6	F: CGCTTCGGCAGCACATATACTA
	R: GCTTCACGAATTTGCGTGTCA
LncRNA F11‐AS1	F: GCTCCAACACACTACTGGCT
	R: AAACGCGCAAAGTTGGTTCA
HBx	F: GGCTCGAGATGGCTGCTAGGCTGTGC
	R: GGCGAATTCAGAAGTCGTCGTCGTCC
NR1I3	F: CATGGGCACCATGTTTGAAC
	R: AGGGCTGGTGATGGATGAA
Bax	F: CAGCTCTGAGCAGATCATGAAGACA
	R: GCCCATCTTCTTCCAGATGGTGAGC
Bcl‐2	F: ATGTGTGTGGAGAGCGTCAACC
	R: TGAGCAGAGTCTTCAGAGACAGCC
PCNA	F: TCTCAGCCATATTGGAGATG
	R: CAGGTACCTCAGTGCAAAAG
GAPDH	F: GGGCTGCTTTTAACTCTGGT
	R: GCAGGTTTTTCTAGACGG

Abbreviations: Bax, Bcl‐2–associated X protein; Bcl‐2, B‐cell lymphoma 2; F, forward; GAPDH, glyceraldehyde 3‐phosphate dehydrogenase; HBx, hepatitis B virus X protein; LncRNA F11‐AS1, long non‐coding RNA F11‐antisense 1; miR‐211‐5p, microRNA‐211‐5p; NR1I3, nuclear receptor constitutive androstane receptor; PCNA, proliferating cell nuclear antigen; R, reverse.

### 2.7 RNA binding protein immunoprecipitation (RIP)

The binding abilities of lncRNA F11‐AS1 and miR‐211‐5p to Argonaute 2 (Ago2) protein were determined using RIP kits (17‐701; Millipore).^15^ A portion (20 μL) of the cell lysate was used as an input, and the other portion was incubated with antibodies for coprecipitation. The magnetic bead‐antibody complex was resuspended in 900 μL RIP immunoprecipitation buffer after washing. The samples were placed on the magnetic seat to obtain a magnetic bead‐antibody complex. The antibodies used for RIP were 5 μg Ago2 antibody (39854; Active Motif) and 5 μg mouse anti‐IgG (ab200699; Abcam). The extracted RNA was purified using the conventional TRIzol method followed by RT‐qPCR assay.

### 2.8 Fluorescence in situ hybridization (FISH)

The subcellular localization of lncRNA F11‐AS1 and its colocalization with miR‐211‐5p were detected using FISH kits (BIS‐P0001; BersinBio, Guangzhou). HepG2.2.15 cells after plasmid delivery in each group were grown in cell slides and rehydrated with 2× sodium citrate buffer for 2‐3 minutes. Then, digoxigenin‐labelled lncRNA F11‐AS1 or miR‐211‐5p–specific probe was added to cell slides with an antagonistic lncRNA F11‐AS1 or miR‐211‐5p probe as the negative control (NC). Hybridization was carried out at 42°C for 16 hours. Finally, the slides were stained with 4′,6‐diamidino‐2‐phenylindole (DAPI) for 5‐10 minutes under dark conditions, and the images were photographed using a confocal laser scanning microscope (Leica Microsystems).

### 2.9 Cell counting kit 8 (CCK‐8)

CCK‐8 assay was performed according to the instructions of the CCK‐8 kit (CK04; Dojindo, Tokyo, Japan). The HepG2.2.15 cells were seeded in a 96‐well plate at a density of 2 × 10^3^ cells per well and incubated with 100 μL MEM‐α complete medium at 37°C with 5% CO_2_ in air. At regular intervals, each well was added with 10 μL CCK‐8 solution at 0th, 24th, 48th and 72nd h after culture, with at least 3 replicates set for each group. Following, the optical density (OD) value was measured at a wavelength of 450 nm after another 4 hours. Finally, the cell growth curve was plotted according to the acquired data.

### 2.10 TdT‐mediated dUTP‐biotin nick end labelling (TUNEL) staining

A total of 5 × 10^7^ cells/mL cells were fixed in 4% paraformaldehyde for 30 minutes at room temperature. The slides were added with 50‐100 μL cell suspension and dried. Afterwards, the cells were incubated with phosphate‐buffered saline (PBS) containing 0.3% Triton X‐100 at room temperature for 5 minutes. Next, TUNEL staining was performed according to the instructions of the TUNEL apoptosis detection kits (C1088; Beyotime Biotechnology Co., Ltd.). The cells were stained with 50 μL TUNEL staining solution at 37°C for 60 minutes avoiding exposure to light. Finally, the sections were mounted by anti‐fluorescence quenching solution and observed under a fluorescence microscope.

### 2.11 RNA pull‐down

The binding of miR‐211‐5p to lncRNA F11‐AS1 and NR1I3 mRNA was detected using Magnetic RNA‐Protein Pull‐Down kits (20164; Pierce). HepG2.2.15 cells were detached and subsequently centrifuged. Then, the precipitate was harvested and lysed with RIP lysis buffer on ice for 2 minutes followed by a centrifugation at 4°C for 10 minutes. The collected supernatant was equally divided into several portions, one of which was used as an input, and stored at −80°C. According to the manufacturer's protocols, miR‐211‐5p, miR‐NC, lncRNA F11‐AS1‐wild type (wt), lncRNA F11‐AS1‐mutant (mut), NR1I3‐wt and NR1I3‐mut were firstly labelled with biotin and then enriched using streptavidin‐labelled magnetic beads. The cells were incubated with the cell lysis buffer at 4°C overnight. The extracted RNA was purified using conventional TRIzol method followed by RT‐qPCR assay.

### 2.12 Scratch test

HepG2.2.15 cells were seeded into a 6‐well plate at a density of 2.5 × 10^4^ cells/cm^2^ and cultured for 24 hours, followed by the removal of the cell culture medium. The straight scratches were made using a 10‐μL sterile pipette. After being washed two times with PBS, the cells were further cultured in MEM‐α complete medium containing 10% FBS. Afterwards, images of each well at 0 and 24 hours post‐scratching were pictured under the inverted microscope, with three replicates set for each group. The width of each scratch was determined using the ImageJ software.

### 2.13 Transwell assay

The invasion ability of HepG2.2.15 cells in each group was assessed using a 24‐well Transwell plate (Corning Incorporated). A total of 2.5 × 10^4^ HepG2.2.15 cells were seeded into the apical chamber coated with 200 mg/mL diluted (1:8) Matrigel (40111ES08; Yeasen Company), and dried overnight under sterile conditions. Then, the basolateral chamber was supplemented with cell culture medium containing serum. After an incubation at 37°C for 24 hours, the non‐invasive cells on the apical chambers were wiped off with cotton. The cells invaded through Matrigel were fixed in 100% methanol for 10 minutes, air‐dried and stained with 0.1% crystal violet. The number of invasive cells was observed under the microscope (200×) (Leica Inc).

### 2.14 Western blot analysis

Western blot analysis was performed according to the previous literature.^16^ Total protein in tissues and cells was extracted using RIPA cell lysis buffer (R0010; Solarbio) containing PMFS, which was then separated through sodium dodecyl sulphate‐polyacrylamide gel electrophoresis (SDS‐PAGE). Then, the protein was transferred onto the polyvinylidene fluoride membrane (PVDF, FFP36; Beyotime Biotechnology Co., Ltd.) and blocked with 5% bovine serum albumin (BSA) at 37°C for 2 hours. Next, the membrane was probed with primary antibodies including mouse polyclonal antibodies to GAPDH (SAB1405848, 1 μg/mL) and NR1I3 (SAB1406904, 1 μg/mL), rabbit polyclonal antibodies to Bax (SAB4502546, 1:1000) and Bcl‐2 (SAB4500005, 1:1000) from Sigma‐Aldrich, and rabbit monoclonal antibody to proliferating cell nuclear antigen (PCNA; ab92552, 1:1000) from Abcam overnight at 4°C. The next day, the membrane was incubated with horseradish peroxidase (HRP)–labelled goat anti‐rabbit IgG (ab6721, 1:20 000) or goat antimouse IgG (ab6789, 1:10 000) from Abcam for 1 h at room temperature. Finally, the protein on the membrane was visualized using an image developing kit (P0020; Beyotime Biotechnology Co., Ltd.), and photographed using a Bio‐Rad gel imaging analysis system (Bio‐Rad). The protein expression was analysed using the ImageJ software (National Institutes of Health).GAPDH was used as an internal control. The experiment was repeated three times.

### 2.15 Dual‐luciferase reporter assay

The bioinformatics prediction online tool (https://cm.jefferson.edu/rna22/Interactive/) was used to predict the binding sites between miR‐211‐5p and lncRNA F11‐AS1 or NR1I3. A dual‐luciferase reporter assay was conducted to verify the potential relationship between miR‐211‐5p and lncRNA F11‐AS1, as well as to ascertain whether NR1I3 was a direct target of miR‐211‐5p. Based on the predicted recognition sites, the 3′untranslated region (3′UTR) fragments of lncRNA F11‐AS1 (lncF11‐AS1 3′UTR‐Wt) and NR1I3 mRNA (NR1I3 3′URT‐Wt) were synthesized, and lncF11‐AS1 3′UTR‐Mut and NR1I3 3′URT‐Mut were also synthesized. The constructed sequences were inserted into the plasmid pmirGLO (E1330; Promega). Using Lipofectamine 2000 (Invitrogen) reagents following the manufacturer's instructions, the pmirGLO‐lncF11‐AS1‐Wt, pmirGLO‐lncF11‐AS1‐Mut, pmirGLO‐NR1I3‐Wt and pmirGLO‐NR1I3‐Mut were cotransfected with miR‐211‐5p mimic or miR‐NC into HEK‐293T cells, respectively. The luciferase activity was determined using a dual‐luciferase assay detection kit (E1910; Promega), and the luminance was measured using a GLoma × 20/20 Luminometer (E5311; Zhongmei Biotechnology Co., Ltd.). The experiments were repeated three times.

### 2.16 Xenograft tumour in nude mice

Specific‐pathogen‐free athymic female BALB/C nude mice (aged 4‐6 weeks, weighing 18‐22 g) purchased from Hunan SLAC Jingda Laboratory Animal Co., Ltd. were enrolled in this study. The plasmid oe‐NC, miR‐211‐5p antagomir, oe‐lncF11‐AS1 and miR‐211‐5p agomir were transfected into HepG2.2.15 cells. Approximately 1 × 10^7^ stably transfected HepG2.2.15 cells were subcutaneously inoculated into the axilla of mice (n = 24, 6 mice each group). After 4 weeks, the mice were euthanatized followed by the removal of the tumour specimens. After weighting and photographing, the tumours were fixed in 4% paraformaldehyde solution with the tumour sections prepared and stored at −80°C for further use. Additionally, the other 24 mice received injections of 2 × 10^6^ stably transfected HepG2.2.15 cells into the tail vein. After 8 weeks, the mice were euthanatized followed by the removal of lung to count the number of metastatic nodules.

### 2.17 Immunocytochemistry and immunohistochemistry

The coverslips of HepG2.2.15 cells were blocked in normal goat serum working solution at 37°C for 10 minutes, followed by incubation with the rabbit polyclonal antibody to NR1I3 (ab186869, Abcam) at 4°C overnight or at 37°C for 1 h. Then, the cells were probed with the secondary antibody, HRP‐labelled goat anti‐rabbit and HRP‐labelled streptavidin (S‐A/HRP) working solution at room temperature for 10 minutes, respectively. Substitution of the primary antibody with PBS served as the NC and the known positive sections as the positive control. The sections were observed under an optical microscope (XSP‐36; Boshida Optical Instrument Co., Ltd.), and 5 high‐power fields were randomly selected for each section, with 100 cells counted for each filed. The positive rate was calculated as follows: the number of positive cells/ the total number of cells.

HBV^+^ or HBV^−^ HCC tissue samples were fixed in 4% paraformaldehyde, paraffin‐embedded and prepared into 5‐μm serial sections. Subsequently, the paraffin‐embedded sections were deparaffinized in xylene, rehydrated with distilled water and then subjected to antigen retrieval with 0.01 mol/L citrate buffer at 95°C for 30 minutes. The sections were then incubated with the primary antibody at 4°C overnight. The following observatory and calculation procedures were performed as previously mentioned.

### 2.18 Nuclear/cytoplasmic fractionation

When cell density reached 1 × 10^6^ cells/mL, the cells were lysed with 200 μL lysis buffer. After centrifugation, the supernatant (cytoplasmic fractions) and the precipitation (nuclear fractions) were added with Buffer SK and anhydrous ethanol, respectively. After centrifugation, the cytoplasmic fractions and nuclear fractions were harvested with elution.

### 2.19 Northern blot analysis

Total RNA content was extracted from each cell line using the TRIzol reagent. The solid sample of RNA was dissolved in the loading buffer, denatured for 10 minutes at 65°C and subjected to 2% formaldehyde gel electrophoresis. The RNA in formaldehyde gel was transferred to a nylon membrane using the syphon method and allowed to stand overnight at room temperature. After being heated at 120°C for 1 hours, the membrane was hybridized and detected using the Dig Northern Start Kit, Dig High Prime DNA Labeling and Detection Starter Kit II. After that, the membrane was added with 2‐3 drops of cytidine 5′‐diphosphate, and the results were visualized using the ChemiDoc XRS + chemiluminescence imaging system (Bio‐Rad) for 30 seconds.

### 2.20 Statistical analysis

Each experiment was repeated at least three times, and the statistics were analysed using the SPSS 21.0 software (IBM Corp.). All data were subjected to analysis of normal distribution and variance homogeneity, and measurement data were expressed by mean ± standard deviation. If data were consistent with normal distribution and even variance, paired *t* test was used for comparisons of data between matched HCC and normal tissues; while unpaired *t* test was applied for comparisons of data between HBV^+^ and HBV^−^HCC tissues or between other two groups. Correlation was analysed using Pearson's correlation coefficient. Comparisons among multiple groups were tested by one‐way analysis of variance (ANOVA), followed by Tukey's post hoc test. A two‐way ANOVA was performed to examine cell viability at different time points, while repeated‐measures ANOVA was conducted for comparisons of time‐based tumour volume measurements. The survival rate of patients was calculated using the Kaplan‐Meier curve and tested with the log‐rank test. A value of *P* < .05 was considered to be indicative of statistical significance.

## 3 RESULTS

### 3.1 Low expression of lncRNA F11‐AS1 is associated with poor prognosis in HBV‐related HCC

**Figure 1:**
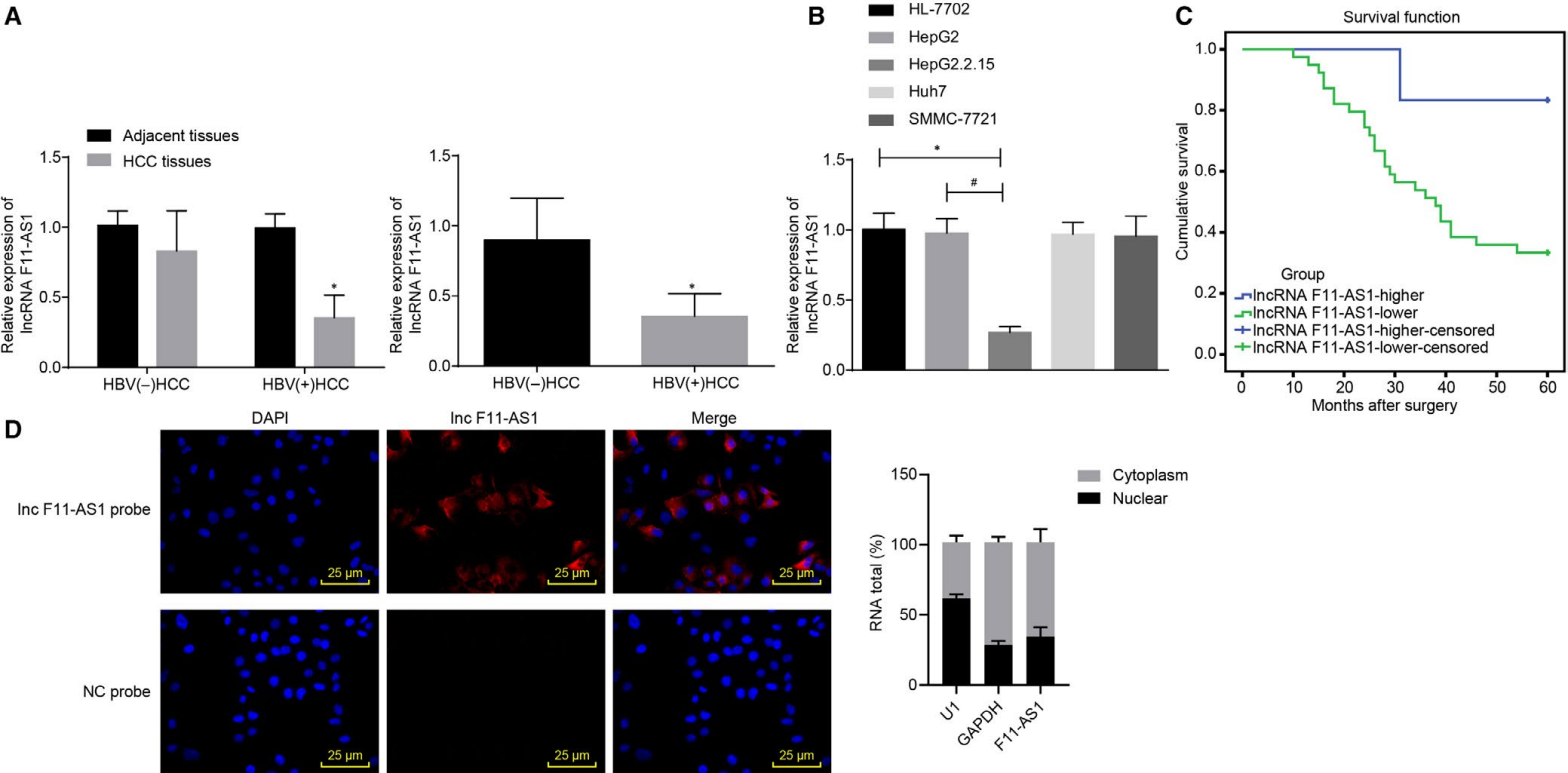
Low expression of lncRNA F11‐AS1 might serve as an indicator of poor prognosis in patients with HBV‐related HCC. A, lncRNA F11‐AS1 expression in HBV^+^/HBV^−^ HCC tissues and corresponding adjacent tissues determined by RT‐qPCR; HBV^+^ HCC tissues = 45, HBV^−^ HCC tissues = 28. B, lncRNA F11‐AS1 expression in HCC cell lines (Huh7, HepG2, HepG2.2.15, SMMC‐7721) and human normal liver cell line HL‐7702 measured by RT‐qPCR. C, Kaplan‐Meier's analysis of survival rates of patients with HBV^+^ HCC regarding higher and lower lncRNA F11‐AS1 expression. D, The localization of lncRNA F11‐AS1 in HepG2.2.15 cells detected by FISH assay and qPCR after nuclear/cytoplasmic fractionation (×400). ^*^ *P* < .05 vs adjacent tissues, HBV^−^ HCC tissues or HL‐7702 cell line; ^#^ *P* < .05 vs the HepG2 cell line. Data were measurement data and expressed by mean ± standard deviation. Comparison of data between matched HCC and normal tissues was analysed by paired *t* test and that between the two groups was analysed by unpaired *t* test, and that among multiple groups was examined by one‐way analysis of variance followed by Tukey's post hoc test

Initially, 73 cases of HBV^+^/HBV^−^ HCC samples and their corresponding adjacent tissues were subjected to RT‐qPCR assay to determine the expression of lncRNA F11‐AS1, and a differential analysis of lncRNA F11‐AS1 expression in HBV^+^ and HBV^−^ tissues was performed. The results revealed that there were no significant differences in lncRNA F11‐AS1 expression between HBV^−^ HCC tissues and corresponding adjacent tissues (^*^ *P* > .05); however, HBV^+^ HCC tissues presented with lower lncRNA F11‐AS1 expression compared to the corresponding adjacent tissues (^*^ *P* < .05). Furthermore, lncRNA F11‐AS1 was poorly expressed in HBV^+^ HCC tissues in comparison with HBV^−^ HCC tissues (^*^ *P* < .05) (Figure 1A).

Additionally, the expression patterns of lncRNA F11‐AS1 in the four HCC cell lines (Huh7, HepG2, HepG2.2.15 and SMMC‐7721) and human normal liver cell line HL‐7702 were also measured by means of RT‐qPCR, aiming to analyse the association between lncRNA F11‐AS1 expression and HBV‐related HCC. It was demonstrated that lncRNA F11‐AS1 expression was not significantly different among the HCC cell lines (Huh7, HepG2 and SMMC‐7721) in comparison with HL‐7702 cells (^*^ *P* > .05). Nevertheless, stably HBV‐expressing HepG2.2.15 cells exhibited down‐regulated expression of lncRNA F11‐AS1 (^*^ *P* < .05), suggesting that there was a high possibility of lncRNA F11‐AS1 being related to HBV (^*/#^ *P* < .05) (Figure 1B).

Considering the differential expressions of lncRNA F11‐AS1 in HBV^+^/HBV^−^ HCC tissues, Kaplan‐Meier's analysis was conducted to analyse the HCC prognosis in regard to lncRNA F11‐AS1 expression. Consequently, a higher survival rate was determined in HBV^+^ HCC patients with a higher lncRNA F11‐AS1 expression than those with lower lncRNA F11‐AS1 expression (^*^ *P* < .05) (Figure 1C). Subsequently, lncRNA F11‐AS1 was found to be primarily localized in the cytoplasm of HepG2.2.15 cells as demonstrated by FISH assay. After the cytoplasmic RNA and nuclear RNA were isolated, the expression of U1 and GAPDH in nucleus and cytoplasm was detected by qPCR. As U1 was known to be mainly expressed in the nucleus, whereas GAPDH was expressed in the cytoplasm, the cytoplasmic and nuclear lncRNA F11‐AS1 were normalized to GAPDH and U1, respectively. It was observed that the expression of lncRNA F11‐AS1 in the cytoplasm was higher than that in the nucleus (^*^ *P* < .05) (Figure 1D). Collectively, the results indicated that poorly expressed lncRNA F11‐AS1 might serve as an indicator of poor prognosis in patients with HBV‐related HCC.

### 3.2 HBx protein inhibits lncRNA F11‐AS1 expression in HCC

**Figure 2:**
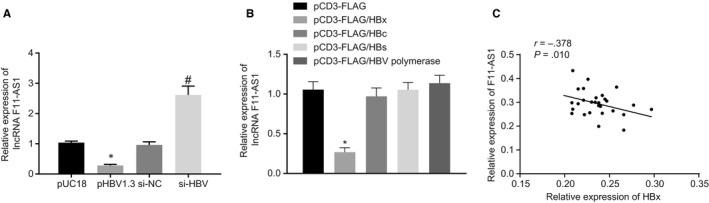
HBx protein contributes to lncRNA F11‐AS1 down‐regulation in HCC cells. A, lncRNA F11‐AS1 expression in HepG2.2.15 cells after plasmid delivery of pUC18‐HBV1.3copy, pUC18, si‐HBx and si‐NC determined by RT‐qPCR; ^*^ *P* < .05 vs HepG2.2.15 cells delivered with pUC18.  # *P* <.05 vs HepG2.2.15 cells delivered with si‐NC. B, lncRNA F11‐AS1 expression in HepG2.2.15 cells after the delivery of pCD3‐FLAG/HBx, pCD3‐FLAG/HBc, pCD3‐FLAG/HBs, pCD3‐FLAG/HBV polymerase and pCD3‐FLAG measured by RT‐qPCR; ^*^ *P* < .05 vs HepG2.2.15 cells delivered with pCD3‐FLAG. C, Correlation between lncRNA F11‐AS1 expression and HBx mRNA expression in patients with HBV^+^ HCC analysed by Pearson's correlation coefficient analysis. Data were measurement data and expressed by mean ± standard deviation. Comparison of data between the two groups was analysed by unpaired *t* test, and that among multiple groups was examined by one‐way analysis of variance followed by Tukey's post hoc test. Pearson's correlation coefficient was applied for Pearson's correlation analysis

lncRNA F11‐AS1 was found to be lowly exhibited in HBV‐related HCC, which might be associated with HBV. To confirm this hypothesis, HBV^−^HepG2.2.15 cells were transiently transfected with pUC18‐HBV1.3copy (pHBV1.3), pUC18, siRNA against HBV (si‐HBV) or si‐NC. Following transfection for 48 hours, the expression of lncRNA F11‐AS1 was determined in the transfection products using RT‐qPCR. As the results depicted in Figure 2A, HepG2.2.15 cells displayed a notable decline in lncRNA F11‐AS1 expression after pHBV1.3 delivery (^*^ *P* < .05). On the contrary, compared with transfection with si‐NC, the lncRNA F11‐AS1 expression was found to be significantly increased following transfection with si‐HBV (^#^ *P* < .05), confirming the inhibition of lncRNA F11‐AS1 caused by HBV.

Next, we shifted our attention to determine how HBV inhibited the expression of lncRNA F11‐AS1 in HCC cells. HepG2.2.15 cells were separately transfected with plasmids expressing HBV‐encoded X, C, S and P proteins including pCD3‐FLAG/HBx, pCD3‐FLAG/HBc, pCD3‐FLAG/HBs and pCD3‐FLAG/HBV polymerase with pCD3‐FLAG serving as the control. Afterwards, the RNA content in the transfected cells was extracted for determination of lncRNA F11‐AS1 expression by RT‐qPCR. As the results described in Figure 2B, significantly decreased expression of lncRNA F11‐AS1 was observed in HepG2.2.15 cells that were delivered with pCD3‐FLAG/HBx compared to those treated with pCD3‐FLAG (^*^ *P* < .05). Meanwhile, the lncRNA F11‐AS1 expression was negatively correlated with HBx mRNA expressions in patients with HBV^+^ HCC according to Pearson's correlation analysis (^*^ *P* < .05).

The HBx gene was the smallest open reading frame of the HBV genome, and its multifunctional proteins are widely involved in viral replication, regulation and expression of gene in host cell.^17^ In recent years, studies focusing on the relationship between HBx protein and lncRNA have confirmed that some lncRNAs are regulated by the HBx protein. In addition, Huang *et al* discovered that lncRNA Dreh was negatively regulated by the HBx protein *via* microarray analyses.^18^ Moreover, Hu *et al* revealed that lncRNA UCA1, which was up‐regulated by the HBx protein, promoted cell growth and tumour formation *via* recruitment of EZH2 and inhibition of p27Kip1/CDK2 signalling.^19^ Although the HBx protein was found to down‐regulate the expression of lncRNA F11‐AS1 in the current study, the underlying mechanism behind HBx protein resulting in this alteration still warrants further investigation. On the whole, the aforementioned findings demonstrated that the HBx protein contributed to lncRNA F11‐AS1 down‐regulation in HCC cells.

### 3.3 lncRNA F11‐AS1 up‐regulation suppresses proliferation, migration and invasion yet induces apoptosis of HBV^+^ HCC cells

**Figure 3:**
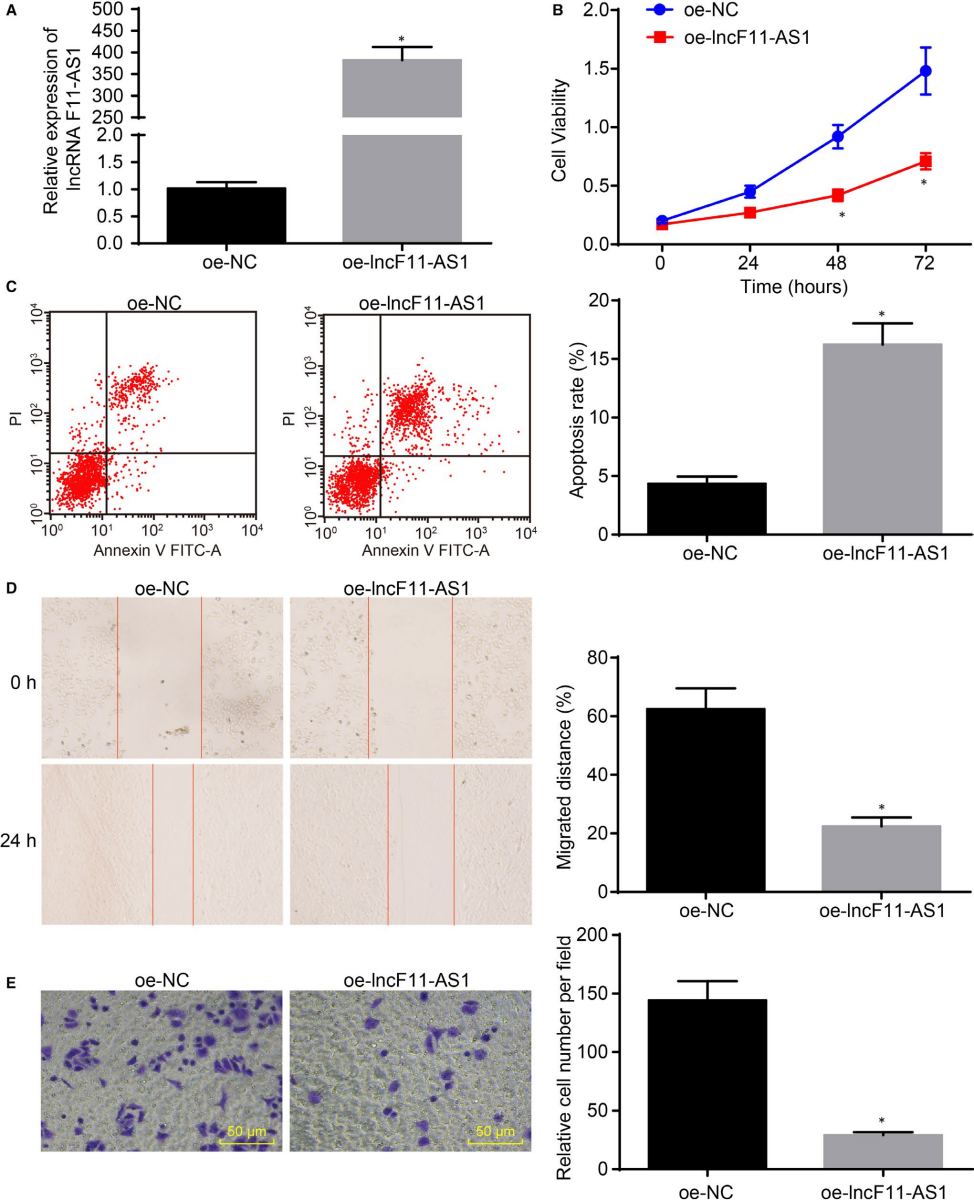
lncRNA F11‐AS1 overexpression inhibits proliferation, migration and invasion of HepG2.2.15 cells while promoting cell apoptosis. HepG2.2.15 cells were transfected with oe‐lncF11‐AS1 or oe‐NC. A, lncRNA F11‐AS1 expression in HepG2.2.15 cells determined by RT‐qPCR. B, The proliferative ability of HepG2.2.15 cells assessed by CCK‐8 assay. C, The apoptosis rate of HepG2.2.15 cells determined by flow cytometry. D, The migration ability of HepG2.2.15 cells measured by scratch test (×40). E, The invasion ability of HepG2.2.15 cells evaluated by Transwell assay (×200). ^*^ *P* < .05 vs the HepG2.2.15 cells transfected with oe‐NC. Data were measurement data and expressed by mean ± standard deviation. Comparison of data between the two groups was analysed by unpaired *t* test

To investigate the regulation of lncRNA F11‐AS1 on cell proliferation, migration, invasion and apoptosis in HBV^+^ HCC progression, we applied thee CCK‐8 assay, scratch test, Transwell assay and flow cytometry, respectively. The HepG2.2.15 cell line stably transfected with oe‐lncF11‐AS1 was successfully constructed according to the verification by RT‐qPCR (^*^ *P* < .05) (Figure 3A). The results showed that overexpression of lncRNA F11‐AS1 resulted in a decrease in proliferative ability of HepG2.2.15 cells (^*^ *P* < .05) (Figure 3B), in addition to significantly promoted apoptosis of HepG2.2.15 cells compared with oe‐NC–transfected cells (^*^ *P* < .05) (Figure 3C). Consistently, lncRNA F11‐AS1 overexpression also contributed to inhibition of migration and invasion of HepG2.2.15 cells (^*^ *P* < .05) (Figure 3D,E). These results supported the conclusion that overexpression of lncRNA F11‐AS1 exerted inhibitory effects on in vitro HCC progression.

### 3.4 lncRNA F11‐AS1 binds to miR‐211‐5p

**Figure 4:**
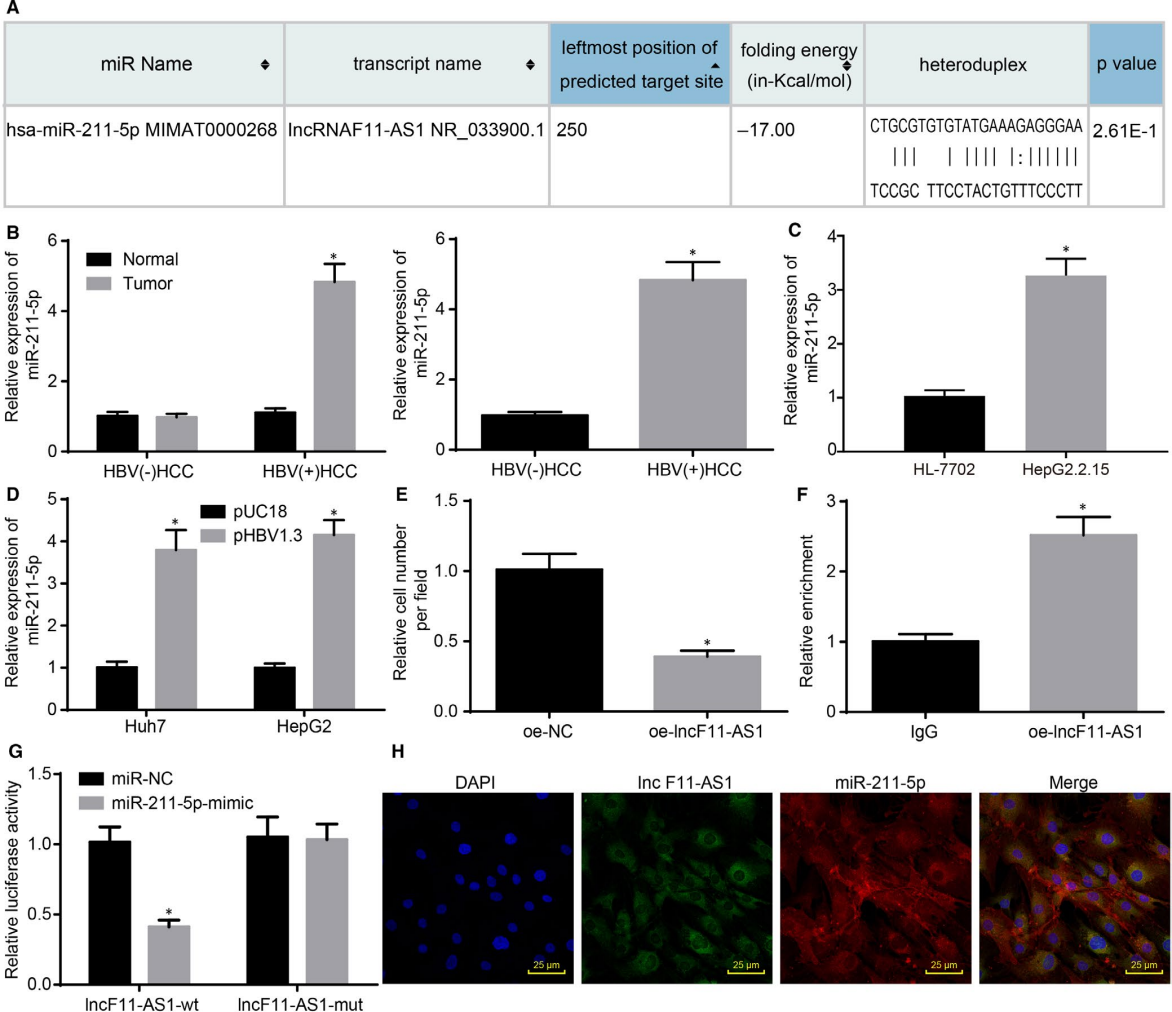
lncRNA F11‐AS1 is capable of binding to miR‐211‐5p. A, The complementary binding sequences between lncRNA F11‐AS1 and miR‐211‐5p predicted by bioinformatics analysis. B, The miR‐211‐5p expression in HBV^+^/HBV^−^HCC tissues and corresponding adjacent tissues; ^*^ *P* < .05 vs adjacent tissues or HBV^−^ HCC tissues; HBV^+^ HCC tissues = 45, HBV^−^ HCC tissues = 28. C, The miR‐211‐5p expression in HepG2 and HepG2.2.15 cells evaluated by RT‐qPCR; ^*^ *P* < .05 vs HepG2 cells. D, The miR‐211‐5p expression in Huh7 and HepG2 cells following transfection with pHBV1.3 or pUC18 assessed by RT‐qPCR; ^*^ *P* < .05 vs Huh7 or HepG2 cells transfected with pUC18. E, The miR‐211‐5p expression in HepG2.2.15 cells following delivery of lncRNA F11‐AS1 overexpressed vectors measured by RT‐qPCR; ^*^ *P* < .05 vs the HepG2.2.15 cells delivered with oe‐NC. F, The binding ability of lncRNA F11‐AS1 to miR‐211‐5p detected by RIP assay; ^*^ *P* < .05 vs HepG2.2.15 cells incubated with IgG. G, The relationship between lncRNA F11‐AS1 and miR‐211‐5p confirmed by dual‐luciferase reporter assay; ^*^ *P* < .05 vs the HEK‐293T cells transfected with NC‐mimic. H, The colocalization of lncRNA F11‐AS1 and miR‐211‐5p in HepG2.2.15 cells detected by FISH assay (×400). Data were measurement data and expressed by mean ± standard deviation. Comparison of data between the two groups was analysed by unpaired *t* test

lncRNA F11‐AS1 was observed to be poorly expressed in HepG2.2.15 cell line, whereas overexpression of lncRNA F11‐AS1 significantly influenced the biological characteristics of HCC cells. Next, we investigated the underlying molecular mechanism of lncRNA F11‐AS1. Based on the prediction results of the RNA22 database (https://cm.jefferson.edu/rna22/Interactive/), lncRNA F11‐AS1 could potentially bind to miR‐211‐5p in HCC (Figure 4A). Interestingly, miR‐211‐5p has also been reported to be differentially expressed in numerous malignancies, as well as to be involved in the regulation of cellular processes such as proliferation, migration and apoptosis.^20^, ^21^, ^22^, ^23^, ^24^ Therefore, the focus of the experiment was shifted to the exploration of the relationship between lncRNA F11‐AS1 and miR‐211‐5p.

The expression patterns of miR‐211‐5p in HBV^±^ HCC tissues and corresponding adjacent tissues were determined by RT‐qPCR, which revealed that there were no significant differences in miR‐211‐5p expression between the HBV^−^ HCC tissues and corresponding adjacent tissues (^*^ *P* > .05). However, a relatively higher expression of miR‐211‐5p was observed in HBV^+^ HCC tissues compared with that in corresponding adjacent tissues (^*^ *P* < .05). Additionally, miR‐211‐5p was found to be highly expressed in HBV^+^ HCC tissues in comparison with the HBV^−^ HCC tissues (^*^ *P* < .05) (Figure 4B). Then, miR‐211‐5p expression and its possible relation to the HBx protein were explored. HBV^+^HepG2.2.15 cells exhibited higher miR‐211‐5p expressions in contrast to HepG2 cells (^*^ *P* < .05) (Figure 4C). Moreover, a significant elevation of miR‐211‐5p was observed in HBV^−^ HepG2 and HBV^−^ Huh7 cells following transfection with pHBV1.3 (^*^ *P* < .05) (Figure 4D), suggesting the inhibition of lncRNA F11‐AS1 caused by HBx protein might affect miR‐211‐5p expression in HCC. Later, down‐regulation of miR‐211‐5p in HepG2.2.15 cells following transfection with oe‐lncF11‐AS1 confirmed this hypothesis (^*^ *P* < .05) (Figure 4E).

Furthermore, RIP assay was applied to measure the binding ability of lncRNA F11‐AS1 to miR‐211‐5p using the Ago2 protein, and the results showed that the lncRNA F11‐AS1–specific probe enriched miR‐211‐5p (^*^ *P* < .05) (Figure 4F). In addition, dual‐luciferase reporter assay was employed to determine the luciferase activity in HEK‐293T cells after cotransfection of lncF11‐AS1‐Wt and lncF11‐AS1‐Mut with miR‐211‐5p mimic or miR‐NC, respectively. It was noted that cotransfection of lncF11‐AS1‐Wt and miR‐211‐5p mimic resulted in a decline in the luciferase activity of HEK‐293T cells, whereas cotransfection of lncF11‐AS1‐Mut and miR‐211‐5p mimic caused no such decrease (^*^ *P* < .05) (Figure 4G). Colocalization of lncRNA F11‐AS1 and miR‐211‐5p was also observed in the cytoplasm of HepG2.2.15 cells using FISH assay (^*^ *P* < .05) (Figure 4H). All the above‐mentioned results demonstrated that lncRNA F11‐AS1 was capable of binding to miR‐211‐5p.

### 3.5 Overexpressed miR‐211‐5p counteracts the tumour suppressive effects of lncRNA F11‐AS1 in HCC

lncRNA F11‐AS1 overexpression conferred significant tumour suppressive effects on HepG2.2.15 cells, which might be achieved through binding to miR‐211‐5p. Hence, we investigated the role of miR‐211‐5p in affecting the biological properties of HCC cells. Therefore, HepG2.2.15 cells were transfected with miR‐211‐5p mimic or NC‐mimic, miR‐211‐5p inhibitor or NC‐inhibitor, or cotransfected with oe‐lncF11‐AS1 and NC‐mimic, or oe‐lncF11‐AS1 and miR‐211‐5p mimic.

**Figure 5:**
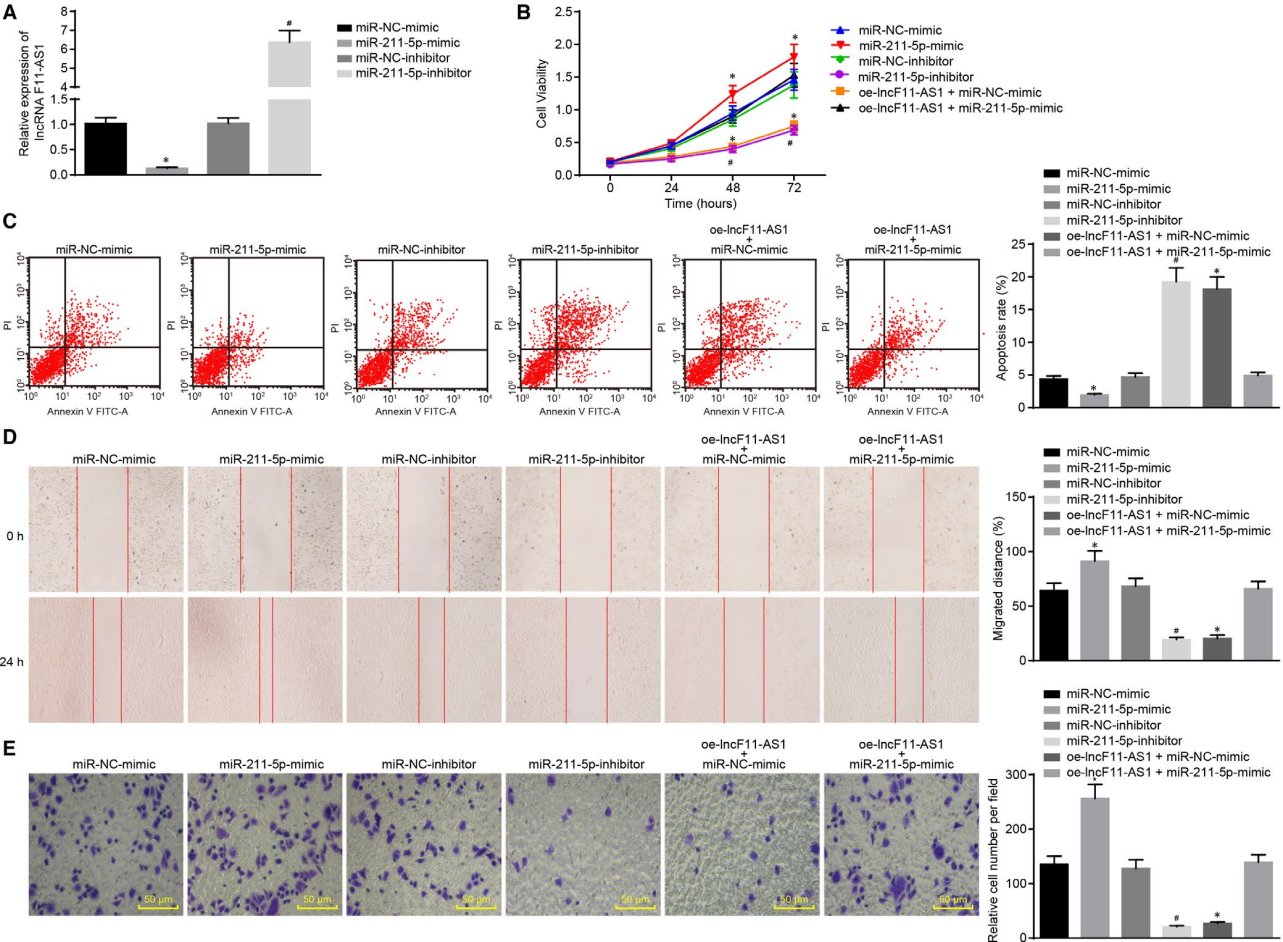
miR‐211‐5p could abolish the anti‐cancer effect of lncRNA F11‐AS1 on HCC. A, The alterations regarding the lncRNA F11‐AS1 expression in HepG2.2.15 cells after transfection with miR‐211‐5p mimic or NC‐mimic, miR‐211‐5p inhibitor or NC‐inhibitor determined by RT‐qPCR. HepG2.2.15 cells were transfected with miR‐211‐5p mimic or NC‐mimic alone miR‐211‐5p inhibitor or NC‐inhibitor or cotransfected with oe‐lncF11‐AS1 and miR‐211‐5p mimic/NC‐mimic together. B, The proliferative ability of HepG2.2.15 cells assessed by CCK‐8 assay. C, The apoptosis rate of HepG2.2.15 cells evaluated by flow cytometry. D, The migration ability of HepG2.2.15 cells measured by scratch test (×40). E, The invasion ability of HepG2.2.15 cells determined by Transwell assay (×200). ^*^ *P* < .05 vs the HepG2.2.15 cells transfected with NC‐mimic; ^#^ *P* < .05 vs the HepG2.2.15 cells delivered with NC‐inhibitor. Data were measurement data and expressed by mean ± standard deviation. Comparison of data among multiple groups was examined by one‐way or two‐way analysis of variance

Subsequently, RT‐qPCR was applied to determine the alterations in gene expression regarding lncRNA F11‐AS1 in HepG2.2.15 cells after transfection. Overexpression of miR‐211‐5p inhibited the expression of lncRNA F11‐AS1, whereas inhibition of miR‐211‐5p resulted in a relative increase in lncRNA F11‐AS1 expression (^*^ *P* < .05) (Figure 5A). The changes in proliferation, apoptosis, migration and invasion of HepG2.2.15 cells caused by different plasmid delivery were assessed separately. Compared with cells transfected with corresponding NC plasmids, cellular up‐regulation of miR‐211‐5p resulted in significantly enhanced cell proliferation and notably decelerated cell apoptosis, while the opposite trends were observed in HepG2.2.15 cells transfected with miR‐211‐5p inhibitor. In addition, it was observed that inhibition of cell proliferation and promotion of cell apoptosis triggered by overexpressing lncRNA F11‐AS1 were reversed after cotransfection with miR‐211‐5p mimic (all ^*/#^ *P* < .05) (Figure 5B,C). Ensuing Transwell assay and scratch test also demonstrated that overexpression of miR‐211‐5p significantly increased migration and invasion abilities of HepG2.2.15 cells, and counteracted the inhibitory effects of lncRNA F11‐AS1 on cell migration and invasion (all ^*/#^ *P* < .05) (Figure 5D,E).

Together, these results supported the conclusion that miR‐211‐5p could abolish the anti‐cancer effect of lncRNA F11‐AS1 on HCC cells.

### 3.6 lncRNA F11‐AS1 up‐regulates NR1I3 expression by competitively binding to miR‐211‐5p

**Figure 6:**
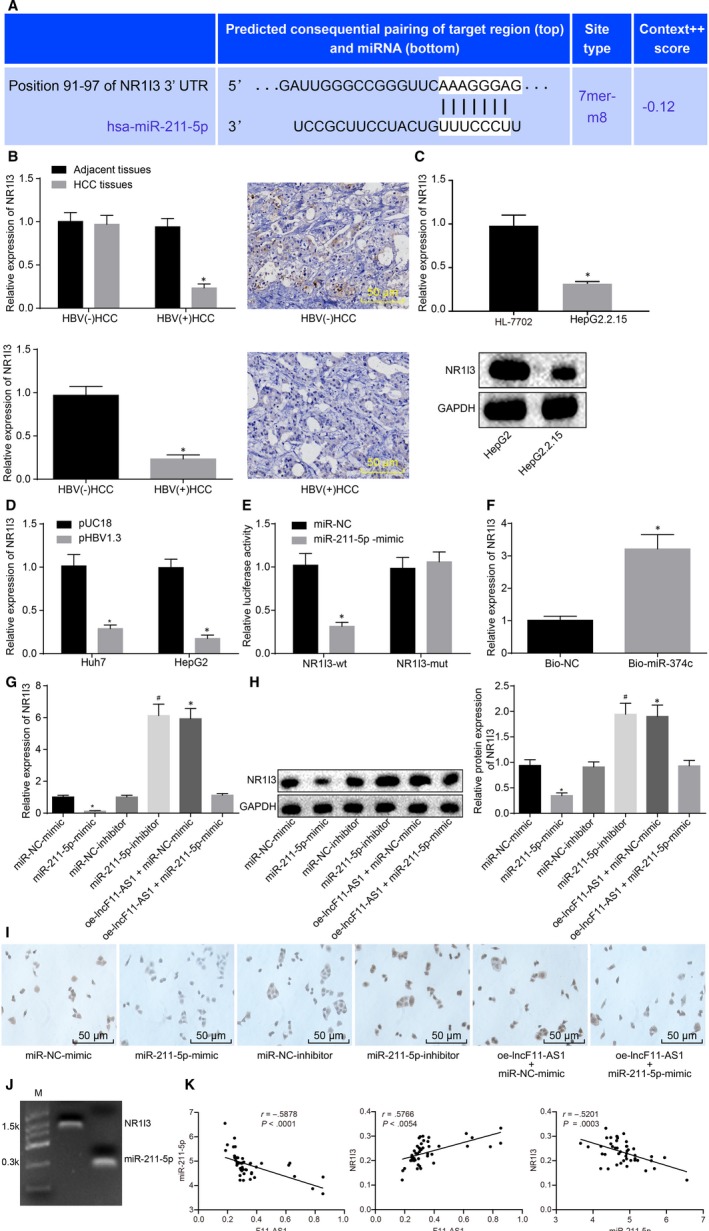
lncRNA F11‐AS1 positively regulates NR1I3 expression *via* competitively binding to miR‐211‐5p. A, The complementary binding sequences between miR‐211‐5p and NR1I3 3’UTR predicted by bioinformatics analysis. B, The NR1I3 expression in HBV^+^/HBV^−^ HCC tissues and corresponding adjacent tissues measured by RT‐qPCR and immunohistochemistry; ^*^ *P* < .05 vs adjacent tissues or HBV^−^ HCC tissues; HBV^+^ HCC tissues = 45, HBV^−^ HCC tissues = 28. C, The mRNA and protein expression of NR1I3 in HepG2 and HepG2.2.15 cells evaluated by RT‐qPCR and Western blot analysis; ^*^ *P* < .05 vs the HepG2 cells. D, The mRNA and protein expression of NR1I3 in Huh7 and HepG2 cells following transfection with pHBV1.3 or pUC18 assessed by RT‐qPCR and Western blot analysis; ^*^ *P* < .05 vs the Huh7 or HepG2 cells transfected with pUC18. E, The relationship between miR‐211‐5p and NR1I3 confirmed by dual‐luciferase reporter assay; ^*^ *P* < .05 vs the HEK‐293T transfected with NC‐mimic. F, The binding ability of miR‐211‐5p to NR1I3 detected by RNA pull‐down; *P < .05 vs the HepG2.2.15 cells incubated with Bio‐NC. G, the mRNA expression of NR1I3 in the transfected HepG2.2.15 cells measured by RT‐qPCR;^*^ *P* < .05 vs the HepG2.2.15 cells transfected with NC‐mimic; ^#^ *P* < .05 vs the HepG2.2.15 cells delivered with miR‐211‐5p inhibitor. H, the protein expression of NR1I3 in the transfected HepG2.2.15 cell determined by Western blot analysis; ^#^ *P* < .05 vs the HepG2.2.15 cells transfected with miR‐211‐5p inhibitor. I, the protein expression of NR1I3 detected by immunocytochemistry. J, the binding status of miR‐211‐5p and NR1I3 detected by Northern blot analysis. K, Pearson's correlation analysis of lncRNA F11‐AS1, miR‐211‐5p and NR1I3 in patients with HBV^+^‐related HCC. Data were measurement data and expressed by mean ± standard deviation. Comparison of data between the two groups was analysed by unpaired *t* test, and that among multiple groups was examined by one‐way analysis of variance followed by Tukey's post hoc test. Pearson's correlation coefficient was used for correlation analysis

Considering the eliminating effects exerted by miR‐211‐5p on tumour suppressive effects of lncRNA F11‐AS1, the focus of the study shifted onto exploration of the possible mechanism of miR‐211‐5p. Subsequently, a binding site between miR‐211‐5p and NR1I3 was found according to bioinformatics analysis (Figure 6A). In addition, NR1I3 has also been documented to exert its regulatory role in breast cancer and colorectal cancer, especially in hepatocarcinogens.^25^, ^26^, ^27^ Then, NR1I3 expression in HBV^+^/HBV^−^ HCC tissues and corresponding adjacent tissues was examined by RT‐qPCR and Western blot analysis. As depicted in Figure 6B, no significant differences were observed regarding NR1I3 expressions in HBV^−^ HCC tissues and corresponding adjacent tissues (^*^ *P* > .05); however, HBV^+^ HCC tissues exhibited relatively lower NR1I3 expression compared with corresponding adjacent tissues (^*^ *P* < .05). Furthermore, compared with HBV^−^ HCC tissues, HBV^+^ HCC tissues displayed significantly low levels of NR1I3 expression (^*^ *P* < .05). As shown in Figure 6C, A lower NR1I3 expression was observed in HBV^+^HepG2.2.15 cells in comparison with HBV^−^HepG2 cells by RT‐qPCR and Western blot analysis (^*^ *P* < .05). Following transfection with pHBV1.3 into HepG2 and Huh7 cell lines, a significant down‐regulation of NR1I3 was determined in both the cell lines (^*^ *P* < .05) (Figure 6D). Subsequent dual‐luciferase reporter gene assay and RNA pull‐down experiments verified that miR‐211‐5p could specifically bind to NR1I3 (^*^ *P* < .05) (Figure 6E,F).

Given that lncRNA F11‐AS1 could bind to miR‐211‐5p, and miR‐211‐5p could specifically regulate NR1I3, we proposed a hypothesis that lncRNA F11‐AS1 might possess the ability to regulate the NR1I3 expression by binding to miR‐211‐5p. Subsequently, HepG2.2.15 cells were transfected with miR‐211‐5p mimic or NC‐mimic, miR‐211‐5p inhibitor or NC‐inhibitor, or cotransfected with oe‐lncF11‐AS1 and NC‐mimic, or oe‐lncF11‐AS1 and miR‐211‐5p mimic, followed by observation of changes in NR1I3 expressions. As depicted in Figure 6G,H, the results of RT‐qPCR and Western blot analysis showed that overexpression of miR‐211‐5p led to a decline in NR1I3 expression (^*^ *P* < .05), whereas inhibition of miR‐211‐5p resulted in an elevation in NR1I3 expression (^#^ *P* < .05). Moreover, cells cotransfected with oe‐lncF11‐AS1 and NC‐mimic displayed significantly increased expression of NR1I3 (^*^ *P* < .05), and those cotransfected with oe‐lncF11‐AS1 and miR‐211‐5p mimic were observed to exert no significant alterations in NR1I3 expression as compared to cells transfected with NC‐mimic (^*^ *P* > .05) (Figure 6G,H). Immunocytochemistry revealed that these results were similar to that observed in HepG2.2.15 cells (Figure 6I). At the same time, the relationship between miR‐211‐5p and NR1I3 was detected by Northern blot analysis, and consequently, the binding of miR‐211‐5p to NR1I3 was substantiated (Figure 6J). We analysed the correlations between the expression of lncRNA F11‐AS1, miR‐211‐5p and NR1I3 in HCC tissues using Pearson's correlation coefficient. The results revealed that the expression of lncRNA F11‐AS1 was negatively correlated with that of miR‐211‐5p, and the same negative correlation was also observed between miR‐211‐5p and NR1I3. On the contrary, the expression of lncRNA F11‐AS1 and NR1I3 was positively correlated (Figure 6K).

These results suggested that NR1I3 was a target gene of miR‐211‐5p and that miR‐211‐5p overexpression inhibited the expression of NR1I3. Either inhibition of miR‐211‐5p or overexpression of lncRNA F11‐AS1 promoted NR1I3 expression in HepG2.2.15 cells. Importantly, lncRNA F11‐AS1 rescued the NR1I3 expression by inhibiting miR‐211‐5p.

### 3.7 lncRNA F11‐AS1/miR‐211‐5p/NR1I3 axis regulates in vitro HCC progression

lncRNA F11‐AS1 was found to regulate NR1I3 expression through acting as a ceRNA of miR‐211‐5p; thus, the influence of this mechanism on biological characteristics of HepG2.2.15 cells was explored in the following experiments. Both lncRNA F11‐AS1 and miR‐211‐5p were observed to influence proliferation and apoptosis of HepG2.2.15 cells; therefore, RT‐qPCR and Western blot analyses were employed to determine the expression of anti‐apoptotic protein Bcl‐2, pro‐apoptotic protein Bax and proliferative‐related protein PCNA.

**Figure 7:**
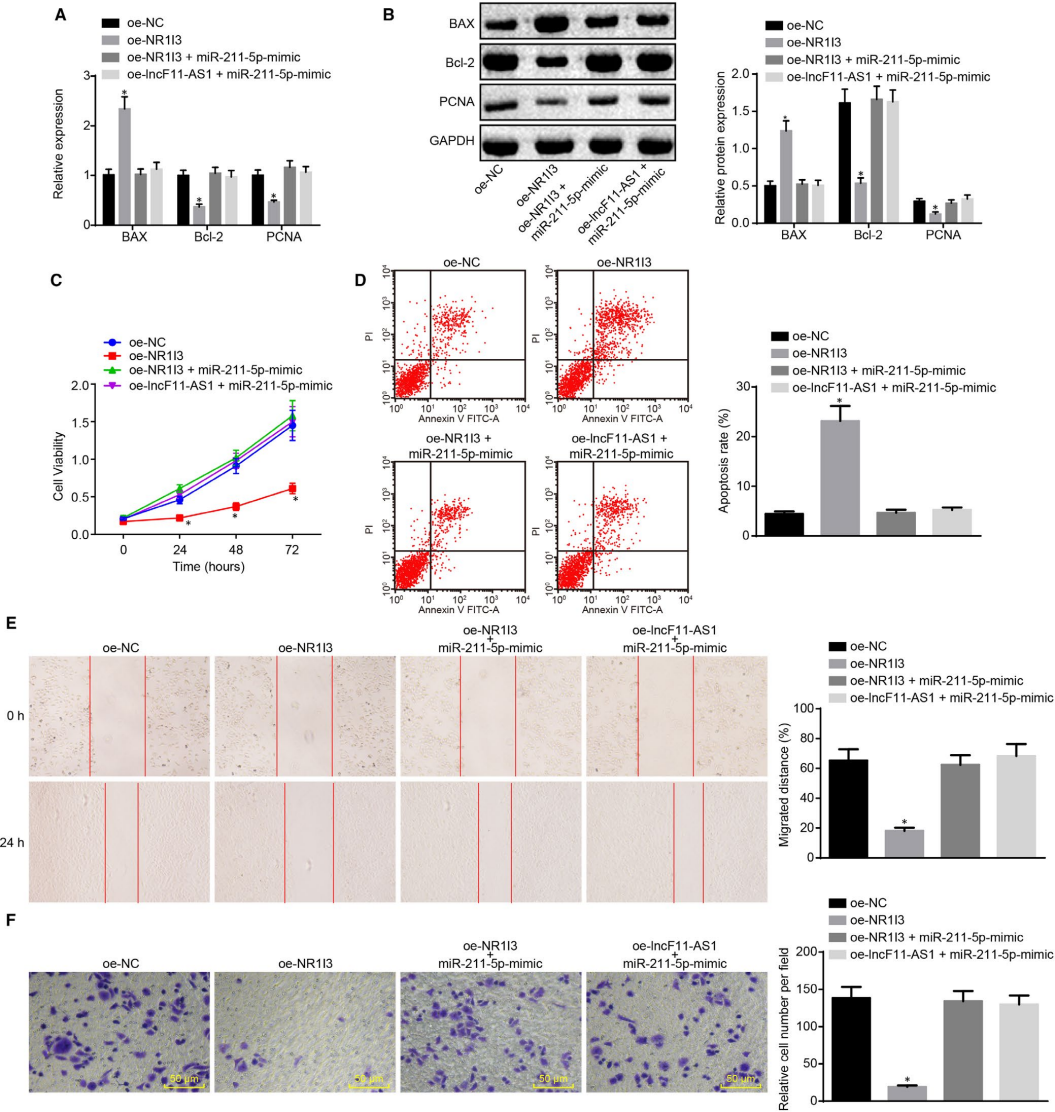
lncRNA F11‐AS1/miR‐211‐5p/NR1I3 axis regulates the proliferation, apoptosis, migration and invasion of HepG2.2.15 cells. A, The mRNA expression of Bax, Bcl‐2 and PCNA in HepG2.2.15 cells after transduction determined by RT‐qPCR. B, The protein expression of Bax, Bcl‐2 and PCNA in HepG2.2.15 cells after transduction measured by Western blot analysis. C, The proliferative ability of HepG2.2.15 cells after transduction assessed by CCK‐8 assay. D, the apoptosis rate of HepG2.2.15 cells after transduction evaluated by TUNEL staining. E, The migration ability of HepG2.2.15 cells after transduction assessed by scratch test (×40). F, The invasion ability of HepG2.2.15 cells after transduction determined by Transwell assay (×200). Data were measurement data and expressed by mean ± standard deviation. ^*^ *P* < .05 vs the HepG2.2.15 cells delivered with oe‐NC. Comparison of data among multiple groups was examined by one‐way or two‐way analysis of variance followed by Tukey's post hoc test

Compared with HepG2.2.15 cells transfected with oe‐NC, those transduced with oe‐NR1I3 had a significant up‐regulation of Bax, whereas those had a notable down‐regulation of Bcl‐2 and PCNA, indicating accelerated cell apoptosis and suppressed cell proliferation (^*^ *P* < .05). This phenomenon was reversed in HepG2.2.15 cells after cotransfection with miR‐211‐5p mimic and oe‐NR1I3, which was similar to the changes in those transfected with oe‐NC (^*^ *P* > .05); there was no remarkable difference concerning those proteins in cells cotransfected with oe‐lncF11‐AS1 and miR‐211‐5p mimic and those transfected with oe‐NC (Figure 7A,B). Corresponding changes in proliferation and apoptosis of HepG2.2.15 cells were more directly reflected in the results of CCK‐8 and flow cytometry (^*^ *P* < .05) (Figure 7C,D). Transwell assay and scratch test revealed that overexpression of NR1I3 significantly reduced the migration and invasion of HepG2.2.15 cells, whereas the inhibitory effects of NR1I3 overexpression on cell migration and invasion were diminished by miR‐211‐5p (^*^ *P* < .05) (Figure 7E,F).

All the above‐mentioned results demonstrated that lncRNA F11‐AS1–impaired miR‐211‐5p–targeted inhibition of NR1I3 could impede the proliferation, apoptosis, migration and invasion of HepG2.2.15 cells, ultimately preventing development and progression of HBV‐related HCC.

### 3.8 lncRNA F11‐AS1 overexpression attenuates tumour growth and metastasis in vivo by inhibiting miR‐211‐5p

The effects of lncRNA F11‐AS1 and miR‐211‐5p on cellular functions of HepG2.2.15 cells have been demonstrated by in vitro experiments; therefore, we employed in vivo mouse experiments to investigate their influences on tumour growth and metastasis. The HepG2.2.15 cells that had been stably transfected with oe‐NC and miR‐211‐5p antagomir, or cotransfected with oe‐lncF11‐AS1 and NC‐agomir, or oe‐lncF11‐AS1 and miR‐211‐5p agomir were subcutaneously administrated into the axilla of mice or intravenously injected into the tail vein, respectively. The mice were euthanatized, followed by measuring the volume of excised tumours and metastatic nodules on the surface of the lungs.

**Figure 8:**
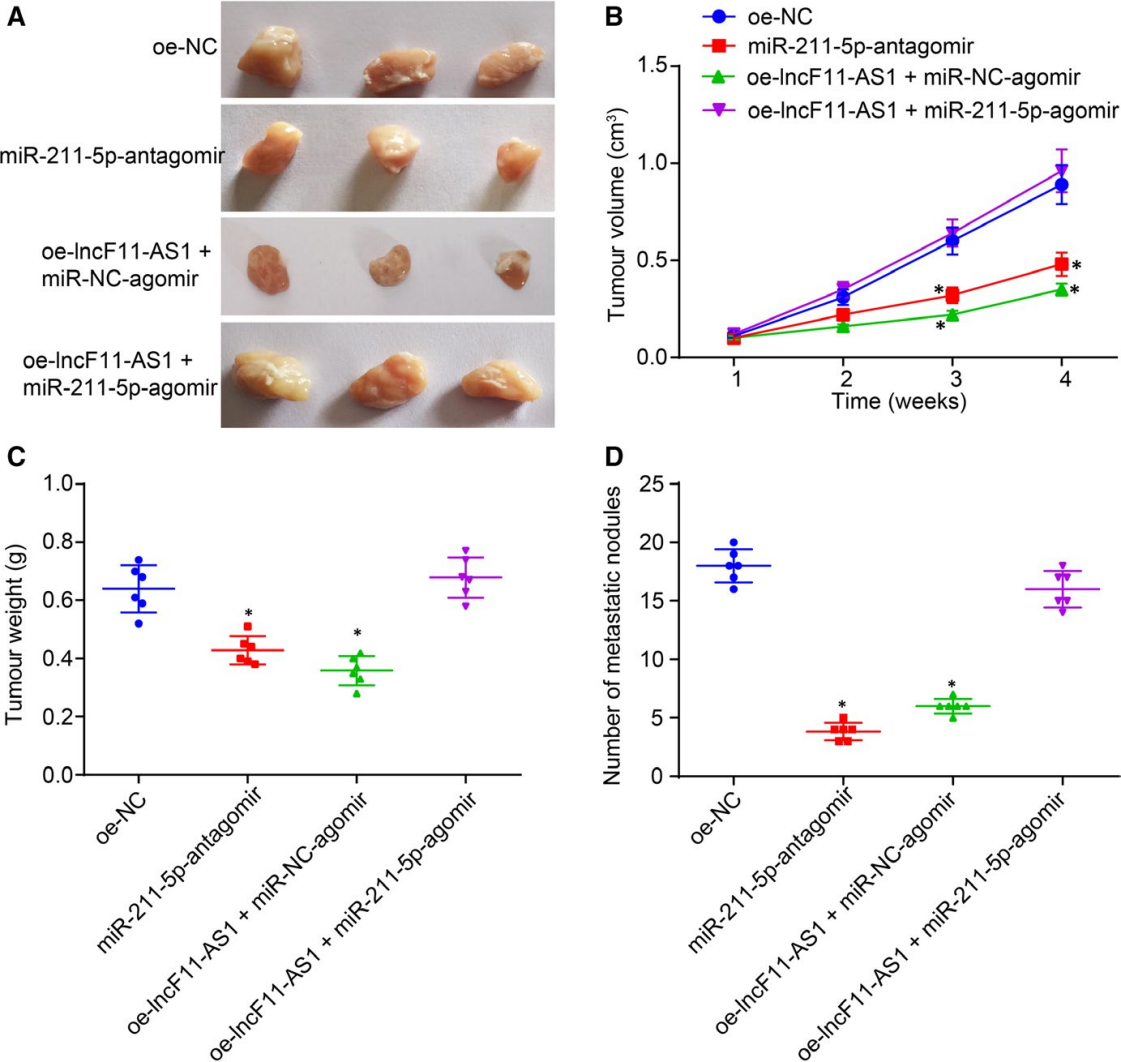
lncRNA F11‐AS1 overexpression and miR‐211‐5p inhibition suppress the tumour growth and metastasis in vivo. Nude mice were injected with HepG2.2.15 cells stably transfected with oe‐NC and miR‐211‐5p antagomir, or cotransfected with oe‐lncF11‐AS1 and NC‐agomir or oe‐lncF11‐AS1 and miR‐211‐5p agomir. A, The photographs of tumour removed from mice. B, Tumour volume of mice at the 1st, 2nd, 3rd and 4th week after injection. C, Statistical analysis of tumour weight. D, The number of metastasis nodules on the surface of lung. ^*^ *P* < .05 vs nude mice injected with the oe‐NC–transfected HepG2.2.15 cells; n = 6. Comparison of data among multiple groups was examined by one‐way analysis of variance or repeated‐measures analysis of variance

Compared with mice receiving an injection of oe‐NC plasmid‐delivered cells, the tumour volume and tumour weight and the number of metastatic nodules were significantly decreased in mice receiving injections of either miR‐211‐5p‐antagomir–transfected cells or oe‐lncF11‐AS1 or NC‐agomir–cotransfected cells (^*^ *P* < .05). There were no significant differences observed in relation to these parameters between mice administrated with miR‐211‐5p‐antagomir–transfected cells and those injected with oe‐lncF11‐AS1 and NC‐agomir–cotransduced cells (^*^ *P* > .05). However, the tumour volume, tumour weight and the number of metastatic nodules were found to be reduced by overexpression of lncF11‐AS1, while they were remarkably increased by overexpression of miR‐211‐5p (^*^ *P* < .05) (Figure 8A–D). All these results led to the conclusion that overexpression of lncRNA F11‐AS1 inhibited the expression of miR‐211‐5p to suppress tumour growth and metastasis in vivo.

## 4 DISCUSSION

The lack of reliable early‐stage diagnostic modalities has resulted in extremely poor prognoses for HCC patients, and therefore, it is trivial to discover more specific and reliable biomarkers for the early detection of HCC to improve prognostic outcomes.^28^ Interestingly, a previous review highlighted the use of lncRNAs as potential markers for cancer diagnosis and prognosis.^29^ In the current study, we aimed to investigate the underlying mechanism of lncRNA F11‐AS1 regulating NR1I3 by binding to miR‐211‐5p, thus serving as a tumour suppressor in HBV‐related HCC progression.

Initially, we uncovered that lncRNA F11‐AS1 was poorly expressed in HBV^+^ HCC tissues and in the HepG2.2.15 cell line, which was correlated with a poor prognosis in HCC patients. Similarly, down‐regulation of lncRNA F11‐AS1 has also been reported in PDAC, and modulation in its expression has been suggested as a new possible biomarker for the diagnosis and treatment of patients with PDAC.^6^ Subsequently, we investigated the cause for triggering lncRNA F11‐AS1 down‐regulation in HCC and discovered that the HBx protein was the primary inhibitor of lncRNA F11‐AS1. The modulation exerted by the HBx protein regarding several cellular processes, such as regulation of transcription and cell cycle progression, has already been demonstrated to be achieved through direct or indirect interaction with host factors related to HCC.^30^ Genetic alterations alone are not enough for the complexity of HBx‐stimulated hepatocarcinogenesis, but the consequent epigenetic changes involving lncRNA expression also contribute to HCC.^31^ HBx protein could act as a negative regulator of lncRNAs to promote HCC progression. For instance, expression of lncRNA down‐regulated by HBx (Dreh) is inversely corrected with the expression of HBx mRNA.^32^ In addition, Dreh is down‐regulated in HBx‐transgenic mice and mouse liver cells expressing HBx, and lncRNA Dreh has the capacity to suppress tumour growth and metastasis of HCC both in vivo and in vitro.^33^ On the other hand, enhanced expression of oncogenic lncRNAs such as DBH‐AS1 and MALAT1 triggered by the suppression of the HBx protein has also been previously demonstrated to promote the progression of HCC.^34^, ^35^ The transcription modulation of HOTAIR by HBV/HBx is likely related to transcription repressive complexes, particularly polycomb repressive complex 2 (PRC2) and LSD1/Co‐REST/HDAC1, which could bind to HOTAIR.^36^ However, detailed mechanism is not defined in this study. In addition, further researches for the underlying mechanism how HBx down‐regulates the expression of lncRNA F11‐AS1 in HCC are necessary in the future.

Furthermore, we explored the role of lncRNA F11‐AS1 as a ceRNA and unfounded that lncRNA F11‐AS1 served a negative regulator of miR‐211‐5p, which was exhibited at high levels in both HBV^+^HCC tissue samples and HepG2.2.15 cells. Recently, lncRNAs have also been demonstrated to be capable of regulating miRNAs at a post‐transcription level through competitively binding to shared miRNAs.^37^ For example, lncRNA KCNQ1OT1 stimulates the proliferation and cisplatin resistance of tongue cancer cells by acting as a ceRNA for miR‐211‐5p.^24^ lncRNA tumour suppressor candidate 7 also acts as a miR‐211 sponge to inhibit colorectal cancer cell proliferation by down‐regulating CDK6.^38^ Principally, our results indicated that lncRNA F11‐AS1 competes with miR‐211‐5p to exert its function in HCC. Later, we also observed that miR‐211‐5p inhibition brought about the same inhibitory effects on HCC cells as lncRNA F11‐AS1 overexpression. Some reports have illustrated that the HBx protein‐induced miRNA expression also shares association with the carcinogenic network of HCC,^39^, ^40^, ^41^ but whether the aberrantly up‐regulated miR‐211‐5p in HCC is directly induced by HBx protein still needed to be investigated.

Additionally, our findings also verified that NR1I3 was targeted and negatively regulated by miR‐211‐5p, and demonstrated low expression of NR1I3 in HBV^+^ HCC tissues and HepG2.2.15 cells. NR1I3 exerts fundamental functions in the modulation of liver homeostasis and tumorigenesis in reaction to xenobiotic stresses.^42^ Previously, decreased expression of NR1I3 due to promoter hypermethylation has been strongly correlated with a decline in cytochrome P450 (CYP) 2C19 expression in HBV‐related HCC tissues, which might play a role in the tumorigenic processes of  HCC.^43^ Remarkably, we found that overexpression of lncRNA F11‐AS1 could up‐regulate the expression of NR1I3 by competitively sponging miR‐211‐5p, and the lncRNA F11‐AS1/miR‐211‐5p/NR1I3 axis might participate in HBV‐related HCC. Previous studies have also shown that the regulatory axis containing lncRNA and miRNA is widely present in multiple diseases. For example, a former study implied that lncRNA MEG3 confers a protective role against congenital intestinal atresia injury via regulation of the miR‐221‐5p/glial cell–derived neurotrophic factor (GDNF) axis, which suppresses hypoxia‐induced intestinal ganglion cell apoptosis.^44^ Moreover, the miR‐211‐5p–mediated inhibition of GDNF was impaired by another lncRNA myocardial infarction–associated transcript, which attenuates neuron apoptosis to alleviate hypoxic‐ischaemic injury.^45^ And both lncRNA F11‐AS1 overexpression and miR‐211 suppression were revealed to increase the expression of Bax protein, while reducing that of Bcl‐2 and PCNA. Consequently, it was suggested that NR1I3 was regulated comprehensively by lncRNA F11‐AS1 and miR‐211‐5p.

**Figure 9:**
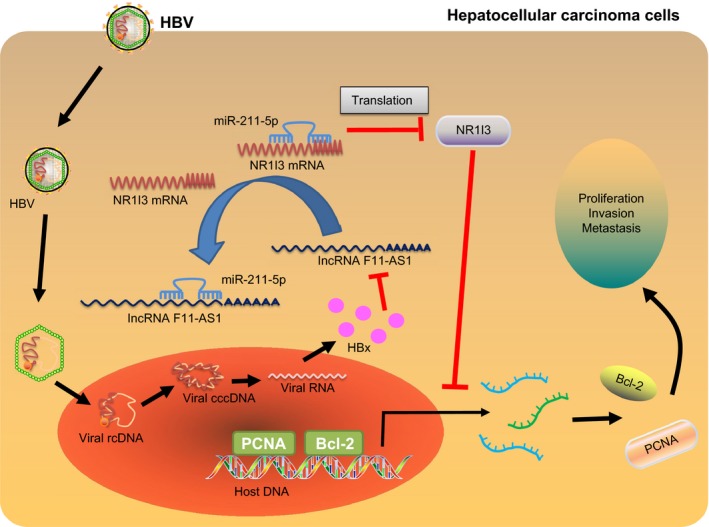
lncRNA F11‐AS1 up‐regulates the expression of NR1I3 *via* miR‐211‐5p to hinder the progression of HBV‐related HCC. In HBV‐related HCC, HBV‐encoded HBx protein inhibited the expression of lncRNA F11‐AS1. lncRNA F11‐AS1 could up‐regulate NR1I3 via binding to miR‐211‐5p, whereas down‐regulation of lncRNA F11‐AS1 caused by HBx protein weakened its ability to bind to miR‐211‐5p, which could target to decrease NR1I3 expression. As a result, the proliferation, migration and invasion of HBV‐related HCC cells were enhanced, yet cell apoptosis was attenuated. Importantly, overexpression of lncRNA F11‐AS1 could enhance the NR1I3 expression by acting as a ceRNA of miR‐211‐5p, ultimately impeding the progression of HBV^+^ HCC

Collectively, these results highlighted that lncRNA F11‐AS1 up‐regulated the expression of NR1I3 via competitively sponging miR‐211‐5p to hinder the progression of HBV‐related HCC (Figure 9). The regulation of lncRNA F11‐AS1/miR‐211‐5p/NR1I3 axis in HBV‐related HCC cell line and in nude mice was preliminarily investigated in the current study. However, the clinical efficacy and future potential use of lncRNA F11‐AS1/miR‐211‐5p/NR1I3 axis in treatment of HBV‐related HCC warrant further studies to improve the overall outcomes of HCC patients.

## CONFLICT OF INTEREST

The authors confirm that there are no conflicts of interest.

## AUTHORS’ CONTRIBUTION

Conception and design: Yibin Deng, Zhongheng Wei, Meijin Huang, Guidan Xu, Wujun Wei, Bin Peng, Shunqiang Nong, Houji Qin; Analysis and interpretation: Wujun Wei, Bin Peng; Data collection: Shunqiang Nong, Houji Qin; Statistical analysis: Meijin Huang, Guidan Xu; Writing the article: Yibin Deng, Zhongheng Wei; Critical revision of the article: Yibin Deng, Zhongheng Wei; Final approval of the article: Yibin Deng, Zhongheng Wei, Meijin Huang, Guidan Xu, Wujun Wei, Bin Peng, Shunqiang Nong, Houji Qin.

## Data Availability

The data sets generated/analysed during the current study are available.
